# Cone Beam Computed Tomography in Oral Cancer: A Scoping Review

**DOI:** 10.3390/diagnostics15111378

**Published:** 2025-05-29

**Authors:** Muhammad Aiman Mohd Nizar, Syed Nabil

**Affiliations:** 1 Department of Craniofacial Diagnostic and Biosciences, Faculty of Dentistry, Universiti Kebangsaan Malaysia, Kuala Lumpur 50300, Malaysia; aimannizar@ukm.edu.my; 2 Department of Oral and Maxillofacial Surgery, Faculty of Dentistry, Universiti Kebangsaan Malaysia, Kuala Lumpur 50300, Malaysia

**Keywords:** cone beam computed tomography (CBCT), oral cancer, head and neck cancer, radiotherapy, surgical navigation systems, complications

## Abstract

**Objectives**: The present scoping review aims to explore and provide an overview of the current applications of cone beam computed tomography (CBCT) in the management of oral cancer. **Methods**: This study was conducted in accordance with the JBI Guidance for Scoping Reviews and reported following the PRISMA Extension for Scoping Reviews (PRISMA-ScR). A systematic search was performed across the following databases: PubMed, OVID, Scopus, Web of Science, and the Cochrane Library to answer the research question: “What are the current applications of CBCT in the perioperative management of patients with oral cancer?”. **Results**: A total of 52 studies met the inclusion criteria. Four major areas of CBCT application in oral cancer were identified: radiotherapy planning and monitoring (25 studies), assessment of bone invasion (16 studies), intraoperative surgical guidance (6 studies), and evaluation of treatment-related complications (5 studies). These findings highlight the diverse but focused use of CBCT across different stages of oral cancer management. **Conclusions**: CBCT is increasingly utilized in the perioperative management of oral cancer, with its application in radiotherapy planning and assessment being the most well-established. However, other uses, such as for surgical navigation and complication assessment, are still emerging, with promising evidence. Further research is needed to expand and validate these applications.

## 1. Introduction

Cone beam computed tomography (CBCT) is an extraoral radiographic scanner designed to produce a three-dimensional image of the maxillofacial bones. It was first introduced at the end of the 20th century mainly for dental implant planning and surgery [1]. Since then, there has been an exponential increase in the adoption of this technology [2]. This is mainly because of its advantages such as its ability to obtain detailed volumetric rendering, its compact size, low radiation dose, rapid image acquisition, and more recently low cost [3]. There are also disadvantages, however, especially when compared to conventional CT, which include the presence of more image artefacts that affect the image quality and also reduced contrast resolution [3].

A recent estimate puts oral cancer, mostly squamous cell carcinomas, as the 16th most common malignant neoplasm, with an incidence of 355,000 cases per year [4]. Oral cancer is often diagnosed at an advanced stage, which affects its prognosis. The delay in diagnosis can be due to a delay of seeking medical care by the patient, a delay in achieving diagnosis, or a delay in initiating treatment following diagnosis [5]. Surgery is still the primary mode of treatment, with radiotherapy and chemotherapy usually used as an adjunct in a more advanced disease [6]. The prognosis of oral cancer is generally better for early-stage oral cancer, with advanced stages, especially cases of nodal involvement, meaning lower rates of survival [7].

While CBCT was initially primarily used for implant planning, its application has now expanded to include more indications. It is now commonly used to obtain images of the dento-maxillofacial region for a variety of indications, including orthodontics, endodontics, periodontics, jaw pathology, paranasal sinuses, maxillofacial trauma, temporomandibular joints, and airway assessment [8,9]. While the use of CBCT in benign pathology in the dento-maxillofacial region is quite established, there is still a lack of discussion on its application in oral malignancies [10,11,12]. In this context, this scoping review aimed to map and summarize the literature data on the application of CBCT in the perioperative management of oral cancers.

## 2. Materials and Methods

### 2.1. Protocol and Registration

This study was conducted following the JBI Guidance for Scoping Reviews [13] recommendations and the PRISMA Extension for Scoping Reviews (PRISMA-ScR). The review protocol was registered in the OSF database under the number [osf.io/gwvf6].

### 2.2. Research Question

Based on the PCC principle—population: patients with oral cancer; concept: the application of CBCT; context: perioperative period of care—the research question for this review is as follows: “What are the current applications of CBCT in the perioperative management of patients with oral cancer?”

### 2.3. Identification of Relevant Studies

Searches were performed in MEDLINE using the PubMed and OVID search engines. Databases such as Web of Science, Scopus, and the Cochrane Library were also utilized in this study. The search strategy was created using MeSH terms, DeCS/MeSH terms, Emtree terms, and other free terms, combined using the Boolean operators “OR” and “AND” (Supplementary Table S1). The electronic search was last updated on 17 March 2025. Manual searches were also performed in reference lists of articles undergoing full-text assessment in the 2nd stage.

### 2.4. Study Selection

Studies were assessed with the pre-defined inclusion and exclusion criteria detailed in Table 1. The retrieved papers were exported to Rayyan^®^ reference manager (https://www.rayyan.ai, accessed on 10 March 2025), and duplicates were removed by the program and manually. The selection process was conducted in two stages. In the first stage, the two authors (M.A.M.N. and S.N.) independently examined titles and abstracts of all identified articles from the electronic search and selected articles relevant to the topic. In the second stage, the full text of the articles selected in the first stage was retrieved, and the two authors independently applied the inclusion/exclusion criteria. Disagreements at both stages were resolved through discussion and a consensus decision between the authors. The reasons for study exclusion in stage 2 of the process was recorded.

### 2.5. Data Extraction

Data were extracted by the first author (M.A.M.N.) from the full text of the accepted articles. When there was ambiguity or unclear information, a discussion between the authors was conducted. If the issue remained unresolved or data were missing, it was recorded as missing data. The accuracy of the extracted data was verified by the second author (S.N.). The data were recorded using a standardized sheet in Microsoft^®^ Excel. Among the selected papers, the following data were extracted: a. publication information (such as year of publication, type of study design, citation received, etc.) and b. the exact application of CBCT. The obtained data were presented descriptively and categorized in tables according to the application of CBCT in oral cancer. Citation data were based on the citations received from the Scopus databases up to 30 April 2025.

## 3. Results

**Figure 1:**
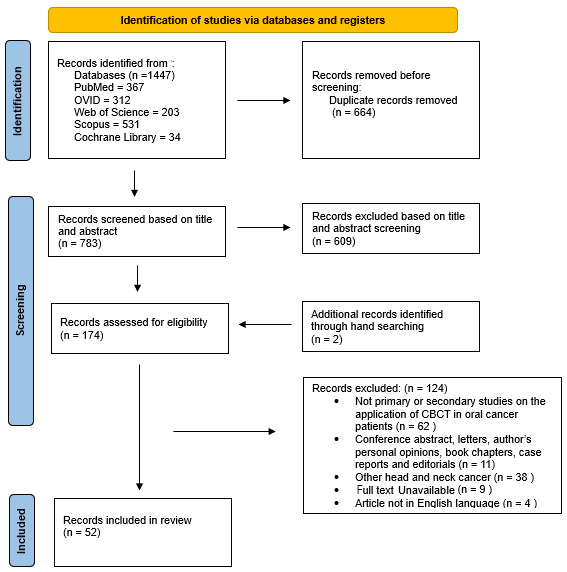
Flowchart summarizing the selection process of papers with a PRISMA-ScR flow diagram.

A total of 1447 titles/abstracts were identified and screened. There were 367 articles identified from PubMed, 312 articles from OVID, 203 from Web of Science, 531 from Scopus, and 34 from the Cochrane Library (Figure 1). After duplicate removal using the Rayyan^®^ reference manager and manually, a total of 783 articles remained. Title and abstract screening of these 783 articles eventually resulted in the exclusion of 609 articles for not being related to the topic. The inclusion and exclusion criteria were then applied to the remaining 174 articles, with two additional articles identified through hand-searching. A total of 124 articles were excluded for not meeting the predefined criteria. The reasons for exclusion were recorded (Supplementary Table S2). In the end, 52 articles were included in this review (Table 2). From the 52 articles included, four general applications of CBCT in oral cancer can be identified. These are its use in radiation treatment, bone invasion assessment, intraoperative surgical guidance, and in assessing complications from the treatment of oral cancer. 

### 3.1. Imaging in Radiotherapy Treatment

This review found that this CBCT application in oral cancer had the most publications, with 24 articles (Table 3). Among the uses of CBCT in oral cancer radiation therapy are verifying patient positioning and assessing tumour changes during the course of treatment. Most of the studies being published on this topic are observational studies, either case–control, prospective, or retrospective studies. The majority of the articles were published within the past 5 years, with the earliest publication on this application being in 2009. Overall, there were 264 citations received for all the articles on this topic, giving a citation rate of 11 citations per article for this application.

### 3.2. Bone Invasion Assessment

Bone invasion assessment is another indication of the use of CBCT in oral cancer. Overall, there are a total of 16 articles on the topic (Table 4). The majority of the use of CBCT is for the assessment of bone invasion in the mandible specifically (n = 11), some assessed bone invasions in both jaws (n = 4), while only one article used CBCT specifically for maxillary bone invasion. The majority of the studies were observational, but there were also three systematic reviews on this topic. Besides having articles with higher levels of evidence, this CBCT application also received the highest citation rate compared to other applications, with 21 citations per article. The first article on this topic, published in 2009, compared CBCT with orthopantomograms (OPGs) in detecting mandibular invasion. Subsequently, more studies were comparing CBCT with other imaging modalities such as conventional CT scans, magnetic resonance imaging (MRI), and single-photon emission CT (SPECT).

### 3.3. Intraoperative Surgical Guidance

There were only six articles that described the application of CBCT for surgical treatment of oral cancer (Table 5). Four of the six articles described its use in assisting the resection of the tumour during surgery. The other two articles used CBCT for sentinel lymph node identification and for intraoperative imaging during minimally invasive cancer surgery. Half of the articles were laboratory experimental studies, while the remaining three were observation clinical studies. The first article on this topic was published in 2014, and the last was published in 2021. The citation rate is the lowest compared to the other applications, with 10 citations/article.

### 3.4. Complication Assessment

CBCT has also been used for the assessment of complications related to oral cancer treatment. We identified five articles of this application (Table 6). The complications that were assessed included osteoradionecrosis, xerostomia, malnutrition, and temporomandibular joint resorption. The study design of the included articles on this application was either prospective or retrospective observational studies. Collectively, the articles on this topic had 19 citations per article. The first article on complication assessment was published in 2009, but in the past 5 years only one further article was related to the oral cancer treatment complication assessment.

## 4. Discussion

CBCT was initially developed for the dento-maxillofacial region, mainly for the planning of dental implants [1]. Prior to CBCT, implant planning was performed two-dimensionally with OPG, which inherently had some limitations due to distortion and magnification and furthermore provided no information on the horizontal bone volume. This inaccuracy was eliminated with the use of CBCT for implant planning, which has now become the “standard of care”, changing the entire landscape of implant treatment in terms of precision and safety [67]. This shift in practice can also be seen in other clinical situations; for example, the most common indication for the use of CBCT in children and adolescents is the assessment of impacted teeth for orthodontics [68]. Endodontics has also seen the assessment of surgical cases with CBCT as the gold standard [69]. Seeing how CBCT has changed a multitude of clinical practices in the dento-maxillofacial region, some adoption or adaptation of CBCT can also be expected in oral cancer management.

This scoping review provides a comprehensive exploration of the applications of CBCT in the management of oral cancer. We identified four general applications of CBCT related to oral cancer. The application with the most publications regarding CBCT use is in radiation therapy. CBCT was incorporated into the linear accelerator (LINAC) in the mid-2000s [70]. This advancement enabled the development of image-guided radiation therapy (IGRT), and the use of CBCT has since become standard practice in modern radiotherapy. It enables quick and convenient imaging during treatment sessions, ensuring precise radiation delivery. The other three applications of CBCT in oral cancer, however, are not as well established. The use of CBCT in detecting bone invasion in oral cancer has been widely discussed in the literature, as reflected by its relatively high citation rate. Most studies evaluate CBCT in comparison with other imaging modalities, highlighting its potential advantages. The remaining two applications of CBCT in oral cancer involve enhancing surgical precision—particularly in achieving predictable surgical margins—and assessing treatment-related complications. Both of these applications are currently in the exploratory stage and require further clinical validation.

CBCT is now widely used in radiation therapy for head and neck cancers, including oral cancers. Prior to the use of CBCT, electronic portal imaging devices (EPIDs) were used for verifying patient setup and field placement. However, they provide limited anatomical detail due to the mostly two-dimensional image. The turn of the century marked the initial application of CBCT in radiation therapy, laying the groundwork for its eventual integration into image-guided treatment workflows [71]. The majority of radiotherapy centres in developed and even developing nations are now equipped with CBCT for rescanning and replanning [72,73]. During radiotherapy, some changes in tumour volume and patient body weight can occur, potentially leading to either overdosage or underdosage of radiation to the target area or surrounding healthy tissues. The use of daily or weekly CBCT enables adaptive radiation therapy (ART), which allows for real-time adjustments to the treatment plan [74]. With ART, radiation delivery can be modified throughout the treatment process, ensuring that the most effective dose reaches the tumour while minimizing exposure to healthy tissues, thereby reducing side effects [74]. It therefore enables tumour delineation, target volume assessment, and detection of inter-fraction setup errors during the treatment phase [25,31,36]. Any dose discrepancies or deviations from the planned doses would give the opportunity for dose recalculation or plan revision [16,23,28]. CBCT can also serve as an essential evaluation tool for assessing the efficacy of immobilization devices [21,34,38]. Overall, the use of CBCT in delivering radiation therapy contributes to greater uniformity in treatment outcomes by balancing technical precision with individualized patient anatomy and tumour characteristics, making it an essential tool in radiotherapy treatment.

CBCT has also been extensively investigated for its application in detecting bone invasion in oral cancer cases. Based on a systematic review conducted in 2018, CBCT was identified as the best imaging modality for detecting bone invasion, compared to other imaging modalities [48]. Other studies have found that CBCT’s diagnostic accuracy in detecting bone invasion is comparable to that of conventional CT scans or MRI [45,47] although its performance is observed to be observer-dependent [53]. In comparison to SPECT, multislice CT, MRI, and conventional CT scans, CBCT exhibits superior sensitivity for detecting bone invasion [54,55]. However, its specificity is considerably lower than that of multislice CT, MRI, and CT [54,55]. Despite its superior accuracy and sensitivity among various imaging techniques, CBCT lacks a soft-tissue window, limiting its utility in assessing soft-tissue involvement, nerve invasion, and tumour staging. Therefore, it is recommended to use CBCT in conjunction with MRI or CT for comprehensive evaluation [44,45]. A conundrum remains in establishing CBCT as a standard practice in oral cancer management. Even if CBCT demonstrates superior sensitivity in detecting bone invasion compared to MRI or conventional CT scans, its routine use may not be justified when patients already undergo MRI or CT as part of initial staging. Unless there is ambiguity regarding bone involvement on the initial imaging, CBCT may be best reserved as an adjunct or confirmatory tool in cases where diagnostic clarity is required. Thus, in our view, CBCT serves better as an adjunct to already established imaging modalities, primarily because MRI or conventional CT—performed as part of routine staging—can often provide similar information. However, if the intent of using CBCT is to replace the OPG, which is routinely required to assess dental conditions prior to radiation therapy, then its use may be warranted as an essential imaging modality in such cases. The decision to use CBCT must also consider the radiation exposure it entails; even though it delivers a lower dose than conventional CT, it still exposes patients to more radiation than panoramic radiography (OPG) and non-radiating modalities such as MRI. Overall, although the capability of CBCT in detecting bone invasion has been demonstrated, it remains a secondary imaging modality reserved for selected cases, given that standard staging imaging—such as MRI or conventional CT—also possesses comparable diagnostic capability in identifying bone involvement.

The use of CBCT for intraoperative imaging in maxillofacial surgery is quite common, especially in cases of orbito-zygomatico-maxillary complex fracture treatment [75]. The implementation of intraoperative CBCT therefore presents several promising strategies to enhance the outcomes of oral cancer surgery. One of the applications of CBCT is assisting the determination of surgical margins to enable more precise resection of tumour boundaries [57,58,60,61]. Ivashchenko et al., for example, suggested the use of CBCT for margin verification immediately after resection [58]. Steybe et al. meanwhile used CBCT for identification of the planned tumour resection that had been marked with a liquid fiducial marker [60]. Polfliet et al. and Weijs et al. utilized CBCT to construct patient-specific resection templates which were used as a guide for resection of the tumour [57,61]. In all these descriptions, it is clear that the use of CBCT involved the need for jawbone resection, highlighting its limitation in soft-tissue delineation for cancer surgery. Besides assisting in obtaining optimum surgical margin, there were also non-clinical laboratory studies assessing the feasibility of applying CBCT in the identification of sentinel lymph nodes and for surgical navigation system application [56,59]. Although these two applications have not yet been translated into clinical practice, there is significant potential to expand the utilization of CBCT in oral cancer surgery.

CBCT has been utilized as a valuable tool in managing surgical and radiation-therapy-related complications following oral cancer treatment. Two of the described uses of CBCT in this aspect are related to osteoradionecrosis (ORN). Brauner et al. used CBCT images and overlaid them with the radiation planning CT images to identify the most suitable sites for implant placement by determining areas of the jaw that had received less radiation [65]. This approach enables the selection of optimal implant sites, thereby avoiding high-dose irradiated regions and ultimately reducing the risk of ORN, thus improving the chances of successful implant placement [65]. In cases where there is a risk for ORN, CBCT has been suggested to be used to monitor the bone healing process following tooth extraction [62]. The low radiation dose provided by CBCT, coupled with its ability to deliver reliable and accurate volumetric assessments, makes it an invaluable tool for monitoring bone healing [62]. In relation to radiotherapy-related complications, prediction of complications, namely xerostomia and malnutrition, can be made using CBCT images obtained during radiotherapy treatment [64,66]. Changes in parotid gland density, as detected through CBCT, have been shown to be associated with the development of long-term xerostomia [64]. CBCT during treatment assessing oral cavity, oropharynx, and oesophagus changes alongside clinical parameters and dose distribution can be used to identify patients who need nutrition supplementation via a feeding tube [66]. In the surgical treatment, CBCT, in combination with MRI, was used to assess TMJ morphology following mandibulotomy, allowing assessment of both the functional and morphological changes [63]. Progress in this application of CBCT has, however, been stagnant, with only a single publication in the past five years supporting this perspective.

The obvious limitation of this review is the omission of CBCT usage in reconstructive surgery of the jaw. CBCT has also emerged as an imaging modality for surgical planning in reconstructing jawbone defects. This application is already well established but was not included, considering the focus of this review was on oral cancers. Most of the articles describing CBCT planned reconstructive surgery are for the reconstruction of the bony defect of the jawbones, i.e., maxilla or the mandible [57,76]. These articles did not delineate cases of oral cancer, osteoradionecrosis, or benign jaw tumours and focused on how CBCT is used in the reconstruction; therefore, they did not meet the predefined criteria to be included in this review. However, a systematic review of computer-assisted surgery (CAS) for mandibular reconstruction found that CBCT is still less commonly used compared to conventional CT scans in computer-assisted surgical planning for jaw reconstruction [77]. It is, however, one of the viable applications of CBCT in oral cancer, specifically in cases needing jawbone reconstruction.

## 5. Conclusions

In conclusion, CBCT has shown promise as a valuable tool in the management of oral cancer, specifically in radiation treatment planning, evaluation of bone invasion, intraoperative navigation, and post-treatment complications. The current body of evidence is still evolving, particularly in its application for surgical guidance and complication assessment, with most studies being small-scale and heterogeneous. Its advantages, including low radiation exposure and cost-effectiveness, make it particularly suitable for repeated imaging and longitudinal monitoring. Nevertheless, imaging modalities such as MRI and conventional CT remain essential for a comprehensive evaluation, especially when assessing soft-tissue involvement.

## Figures and Tables

**Table 1:** Inclusion and exclusion criteria.

Inclusion criteria	a. Any primary or secondary studies on the application of CBCT in oral cancer patients. b. Only malignant growth from the lips or oral cavity (tongue, buccal mucosa, floor of mouth, upper and lower gingiva, retromolar trigone, and hard palate) [14]. c. Articles published after the year 1998 (i.e., after 1st publication on CBCT for the dento-maxillofacial region). d. Article in the English language.
Exclusion criteria	a. Conference abstracts, letters, author’s personal opinions, book chapters, case reports, and editorials. b. Other head and neck cancers such as those of the glottis, larynx, hypopharynx, nasopharynx, ethmoid, nose, paranasal sinuses, or salivary glands are excluded. c. Full text unavailable.

**Table 2:** Summary of utilization of CBCT in oral cancer.

Indication	Number of Articles	Highest Level of Evidence	Citation Received (up to April 2025)	Citation per Article	Date of First Publication on Topic	Publications Number Since 2020
Imaging in radiation treatment	25	Case–control	483 citations	19.3	2009	16
Bone invasion assessment	16	Systematic review	334 citations	20.9	2009	7
Intraoperative surgical guidance	6	Prospective observational study	60 citations	10	2014	3
Complication assessment	5	Prospective observational study	96 citations	19.2	2009	1

**Table 3:** Summary of studies regarding the use of CBCT during radiation treatment.

Author	Title	Year	Study Design	Objective of Study	Citations
Cheung et al. [15]	Dose recalculation and the Dose-Guided Radiation Therapy (DGRT) process using megavoltage cone-beam CT	2009	PS	To demonstrate the process of performing dose recalculation on megavoltage CBCT images	41
Den et al. [16]	Daily Image Guidance With Cone-Beam Computed Tomography for Head-and-Neck Cancer Intensity-Modulated Radiotherapy: A Prospective Study	2010	PS	To report on the use of daily kilovoltage CBCT to evaluate the inter-fraction and residual error motion of patients undergoing intensity-modulated radiotherapy for head and neck cancer	219
Yan et al. [17]	Expected treatment dose construction and adaptive inverse planning optimization: Implementation for offline head and neck cancer adaptive radiotherapy	2013	RS	To evaluate expected treatment dose for adaptive inverse planning optimization and evaluate it on head and neck cancer adaptive treatment modification using daily CBCT images	23
Hermans et al. [18]	Weekly kilovoltage cone-beam computed tomography for detection of dose discrepancies during (chemo)radiotherapy for head and neck cancer	2015	RS	To evaluate discrepancies between planned and actually delivered radiation doses in head and neck patients and identify predictive factors using weekly CBCT images	9
Hvid et al. [19]	Accuracy of software-assisted contour propagation from planning CT to cone beam CT in head and neck radiotherapy	2016	RS	To determine the accuracy of automated contours compared to manually corrected contours using daily CBCT images	23
Hofmaier et al. [20]	Multi-criterial patient positioning based on dose recalculation on scatter-corrected CBCT images	2017	RS	To evaluate the feasibility and potential advantages of dose-guided patient positioning based on dose recalculation on scatter-corrected CBCT images	19
Norfadilah et al. [21]	Immobilisation precision in VMAT for oral cancer patients	2017	PS	To evaluate the positioning reproducibility of immobilization devices during oral cancer radiotherapy by using CBCT images	2
Brivio et al. [22]	Selection of head and neck cancer patients for adaptive re-planning of radiation treatment using kV-CBCT	2018	RS	To develop a CBCT-based method for the selection of head and neck cancer patients that may require adaptive re-planning	5
McCulloch et al. [23]	Predictive Models to Determine Clinically Relevant Deviations in Delivered Dose for Head and Neck Cancer	2019	RS	To develop a predictive model based on the radiation delivered dose deviations assessed by CBCT	17
Wei et al. [24]	Quantifying the impact of optical surface guidance in the treatment of cancers of the head and neck	2020	RS	To assess the setup accuracy and time for surface-guided radiation therapy using CBCT to verify patient positioning	10
Hague et al. [25]	Inter-fraction robustness of intensity-modulated proton therapy in the post-operative treatment of oropharyngeal and oral cavity squamous cell carcinomas	2020	RS	To evaluate the robustness of an inter-fraction multifield optimized with the use of CBCT in regard to radiation clinical target volume coverage	15
Figen et al. [26]	Radiotherapy for Head and Neck Cancer: Evaluation of Triggered Adaptive Re-planning in Routine Practice	2020	RS	To evaluate the rate of re-planning and determine the factors for a re-plan by assessing anatomical changes with CBCT	32
Morgan et al. [27]	Exploratory ensemble interpretable model for predicting local failure in head and neck cancer: the additive benefit of CT and intra-treatment cone-beam computed tomography features	2021	CCS	To analyse local failure in head and neck cancer using images from CT and CBCT to develop a model for predicting local failure	16
De Ornelas et al. [28]	CBCT-Based Adaptive Assessment Workflow for Intensity Modulated Proton Therapy for Head and Neck Cancer	2021	RS	To explore CBCT use in calculating the proton dose for adaptive radiotherapy in intensity-modulated proton therapy for head and neck cancer	15
Kanehira et al. [29]	Comparisons of normal tissue complication probability models derived from planned and delivered dose for head and neck cancer patients	2021	RS	To compare planned radiation dosage with the delivered dose as estimated from daily CBCT images	2
Shinde et al. [30]	Quantification of 6D inter-fraction tumour localisation errors in tongue and prostate cancer using daily KV-CBCT for 1000 IMRT and VMAT treatment fractions	2022	RS	To evaluate the use of CBCT in minimizing tumour localization errors	0
Rachi et al. [31]	Development of Machine-Learning Prediction Programs for Delivering Adaptive Radiation Therapy With Tumor Geometry and Body Shape Changes in Head and Neck Volumetric Modulated Arc Therapy	2023	RS	To use CBCT to identify objective indicators for cases needing adaptive radiation therapy and develop machine-learning prediction programs	1
All et al. [32]	In Silico Analysis of Adjuvant Head and Neck Online Adaptive Radiation Therapy	2024	PS	To evaluate the benefits of single versus weekly adaptive radiation therapy in head and neck cancer by assessing planning target volume changes using CBCT	0
Sharma et al. [33]	X-ray and MR Contrast Bearing Nanoparticles Enhance the Therapeutic Response of Image-Guided Radiation Therapy for Oral Cancer	2023	AS	To demonstrate the use of theranostic nanoparticles in image-guided radiation therapy planning, utilizing CBCT for tumour delineation and radiation beam arrangement	4
Jung et al. [34]	Novel tongue-positioning device to reduce tongue motions during radiation therapy for head and neck cancer: Geometric and dosimetric evaluation	2023	PS	To verify the position of the tongue during radiation therapy for oral cancer	0
Bobic et al. [35]	Large anatomical changes in head-and-neck cancers—A dosimetric comparison of online and offline adaptive proton therapy	2023	RS	To evaluate an online adaptive workflow intensity-modulated proton therapy versus full offline re-planning with CBCT used to assess anatomical changes	17
Jain et al. [36]	A prospective study to assess and quantify the setup errors with cone-beam computed tomography in head-and-neck cancer image-guided radiotherapy treatment	2023	PS	To assess and quantify the setup errors with CBCT in head and neck cancer image-guided radiotherapy treatment	3
Håkansson et al. [37]	CBCT-based online adaptive radiotherapy for head and neck cancer-dosimetric evaluation of first clinical experience	2023	PS	To assess the dosimetric difference between the daily adapted plans and the original plan with daily CBCT	5
Mail et al. [38]	Evaluation of positioning accuracy in head-and-neck cancer treatment: A cone beam computed tomography assessment of three immobilization devices with volumetric modulated arc therapy	2024	RS	To assess the precision and repeatability of the daily patient positioning for three immobilization devices used for patients undergoing radiation using CBCT	0
Blumenfeld et al. [39]	Real world clinical experience using daily intelligence-assisted online adaptive radiotherapy for head and neck cancer	2024	PS	To evaluate the initial clinical experience of daily adaptive radiotherapy for patients with head and neck cancer using an online adaptive platform with intelligence-assisted workflows with daily CBCT	5

PS: prospective observational study; RS: retrospective observational study; CCS: case–control study; AS: animal study.

**Table 4:** Summary of studies regarding the use of CBCT in bone invasion assessment.

Authors	Title	Year	Study Design	Objective of Study	Citations
Momin et al. [40]	Diagnostic accuracy of cone-beam CT in the assessment of mandibular invasion of lower gingival carcinoma: Comparison with conventional panoramic radiography	2009	RS	To compare the diagnostic accuracy of CBCT versus panoramic radiography in assessing mandibular invasion by oral cancer	47
Hendrikx et al. [41]	Cone-beam CT in the assessment of mandibular invasion by oral squamous cell carcinoma: results of the preliminary study.	2010	RS	To compare the diagnostic value of CBCT in assessing mandibular invasion by oral cancer with panoramic radiography, MRI, and histological examination	59
Dreiseidler et al. [42]	A comparison of multislice computerized tomography, cone-beam computerized tomography, and single photon emission computerized tomography for the assessment of bone invasion by oral malignancies	2011	PS	To compare the performance of CBCT with conventional CT and SPECT in the detection of bone invasion from oral cancer	41
Uribe et al. [43]	Accuracy of imaging methods for detection of bone tissue invasion in patients with oral squamous cell carcinoma	2013	SR	To examine the available evidence on the diagnostic accuracy of imaging methods for the detection of mandibular bone tissue invasion by squamous cell carcinoma	71
Hakim et al. [44]	Imaging of mandible invasion by oral squamous cell carcinoma using computed tomography, cone-beam computed tomography and bone scintigraphy with SPECT	2014	CCS	To compare the performances in detecting bone invasion of the mandible in oral cancer between CT, CBCT, and bone scintigraphy with SPECT	28
Linz et al. [45]	Performance of cone beam computed tomography in comparison to conventional imaging techniques for the detection of bone invasion in oral cancer	2015	RS	To compare the performance of CBCT with OPG radiographs, conventional CT, MRI, and SPECT in the detection of bone invasion from oral cancer	26
Czerwonka et al. [46]	High-resolution cone-beam computed tomography for assessment of bone invasion in oral cancer: Comparison with conventional computed tomography	2017	PS	To compare the sensitivity and specificity to detect mandibular invasion with conventional CT among patients with oral cancer	12
Bombeccari et al. [47]	Accuracy of the Cone Beam Computed Tomography in the Detection of Bone Invasion in Patients with Oral Cancer: A Systematic Review	2019	SR	To examine the available evidence on the accuracy of CBCT compared with other imaging techniques to detect the degree of invasion of the bone tissue in oral cancer	16
Qiao et al. [48]	Performance of different imaging techniques in the diagnosis of head and neck cancer mandibular invasion: A systematic review and meta-analysis	2018	SR	To examine the available evidence on the diagnostic efficacy of the different imaging techniques for mandibular invasion by head and neck cancer	18
Shree et al. [49]	CBCT-based active contour segmentation of bone invasion in oral squamous cell carcinoma—A preliminary retrospective study	2020	RS	To compare the volumetric analysis by manual and semiautomatic active contour segmentation in oral cancer with frank mandibular bone invasion	0
Abhinaya et al. [50]	Evaluation of osseous changes in oral carcinoma-2D versus 3D imaging	2020	RS	To compare osseous changes using OPG radiographs and CBCT in oral cancer cases	0
Wang et al. 2021 [51]	Accuracy of cone-beam computed tomography for the evaluation of mandible invasion by oral squamous cell carcinoma	2021	ELS	To evaluate the accuracy of CBCT to detect mandibular erosion or invasion with spiral CT among patients with oral cancer	6
Shenoy et al. [52]	Evaluation of patterns of mandibular bone invasion in CBCT of patients with oral squamous cell carcinoma: A descriptive study	2022	RS	To evaluate the patterns of mandibular invasion of oral cancer in CBCT images	0
Slieker et al. [53]	Value of cone beam computed tomography for detecting bone invasion in squamous cell carcinoma of the maxilla	2022	RS	To determine the diagnostic value of CBCT in detecting bone invasion in cancer of the maxilla	4
Straub et al. [54]	Performance of cone-beam computed tomography (CBCT) in comparison to conventional computed tomography (CT) and magnetic resonance imaging (MRI) for the detection of bone invasion in oral squamous cell cancer (OSCC): a prospective study	2024	PS	To compare the ability to detect bone erosion or invasion with conventional CT and MRI among patients with oral cancer	5
Neal et al. [55]	The utility of cone-beam computed tomography and multislice computed tomography scan for the evaluation of invasion versus erosion by mandibular squamous cell carcinoma as viewed on medical PACS	2024	RS	To compare the utility for detecting mandibular erosion or invasion with conventional CT among patients with oral cancer	1

PS: prospective observational study; RS: retrospective observational study; CCS: case–control study; SR: systematic review; ELS: experimental laboratory study.

**Table 5:** Summary of studies regarding the use of CBCT for intraoperative surgical guidance.

Author	Title	Year	Study Design	Objective of Study	Citations
Daly et al. [56]	A surgical navigation system for non-contact diffuse optical tomography and intraoperative cone-beam CT	2014	ELS	To assess the feasibility of a non-contact diffuse optical tomography system for multimodal imaging with intraoperative CBCT during minimally invasive cancer surgery	5
Weijs et al. [57]	Accuracy of virtually 3D planned resection templates in mandibular reconstruction	2016	PS	To evaluate the accuracy of prefabricated surgical resection templates using CBCT used in mandibular segmental resections	37
Ivashchenko et al. [58]	Intraoperative verification of resection margins of maxillary malignancies by cone-beam computed tomography	2019	PS	To assess the feasibility of intraoperative imaging during maxillectomy to verify the planned resection margins	5
Muhanna et al. [59]	Sentinel lymph node mapping using ICG fluorescence and cone beam CT—a feasibility study in a rabbit model of oral cancer	2020	AS	To assess the technical feasibility of intraoperative indocyanine green (ICG)-based near-infrared (NIR) fluorescence imaging and CBCT for sentinel lymph node identification during head and neck surgery	8
Steybe et al. [60]	Intraoperative marking of the tumour resection surface for improved radiation therapy planning in head and neck cancer: preclinical evaluation of a novel liquid fiducial marker	2020	AS	To assess the feasibility of using CBCT as part of intraoperative marking of the tumour resection surface in oral cancer patients	3
Polfliet et al. [61]	Registration of magnetic resonance and computed tomography images in patients with oral squamous cell carcinoma for three-dimensional virtual planning of mandibular resection and reconstruction	2021	RS	To evaluate an automated method to perform image registration of MRI and CBCT to be integrated for the virtual planning of mandibular resection margins in oral cancer	2

PS: prospective observational study; RS: retrospective observational study; AS: animal study; ELS: experimental laboratory study.

**Table 6:** Summary of studies regarding the use of CBCT as a tool to assess complications in oral cancers.

Author	Title	Year	Study Design	Objective of Study	Citations
Agbaje et al. [62]	Bone healing after dental extractions in irradiated patients: a pilot study on a novel technique for volume assessment of healing tooth sockets	2009	PS	To assess socket healing following extraction in irradiated jaws	22
Al-Saleh et al. [63]	Three-dimensional morphological changes of the temporomandibular joint and functional effects after mandibulotomy	2017	PS	To assess TMJ morphological changes following mandibulotomy compared to the transoral approach	12
Rosen et al. [64]	Early Changes in Serial CBCT-Measured Parotid Gland Biomarkers Predict Chronic Xerostomia After Head and Neck Radiation Therapy	2018	RS	To assess the applicability of serial CBCT images to predict the occurrence of chronic xerostomia	47
Brauner et al. [65]	Implant placement in oral squamous cells carcinoma patients treated with chemoradiotherapy: “Sapienza Head and Neck Unit” clinical recommendations	2019	PS	To prevent the occurrence of osteoradionecrosis following dental implant by blending radiation dosimetry planning CT scan with CBCT	4
Dohopolski et al. [66]	Use of deep learning to predict the need for aggressive nutritional supplementation during head and neck radiotherapy	2022	RS	To assess the use of CBCT as an adjunct to predict the need for aggressive nutrition supplementation to prevent malnutrition	11

PS: prospective observational study; RS: retrospective observational study.

## Data Availability

The original contributions presented in this study are included in the article/Supplementary Materials. Further inquiries can be directed to the corresponding author.

## Supplementary Materials

The following supporting information can be downloaded at: https://www.mdpi.com/article/10.3390/diagnostics15111378/s1. Table S1: Search strategies for online databases. Table S2: Excluded articles based on inclusion and exclusion criteria.

## Abbreviations

The following abbreviations are used in this manuscript:

CBCT	Cone beam computed tomography
OPG	Orthopantomogram
MRI	Magnetic resonance imaging
TMJ	Temporomandibular joint
CT	Computed tomography
IGRT	Image-guided radiation therapy
SPECT	Single-photon emission CT
ART	Adaptive radiation therapy
LINAC	Linear accelerator
EPIDs	Electronic portal imaging devices
CAS	Computer-assisted surgery
ORN	Osteoradionecrosis
