# Supplementary material for: Cone Beam Computed Tomography in Oral Cancer: A Scoping Review
Source: Diagnostics (Basel). 2025 May 29;15(11):1378. doi: 10.3390/diagnostics15111378 (PMC12155499; doi:10.3390/diagnostics15111378)
Supplement: Supplementary file 1 [file diagnostics-15-01378-s001.zip › diagnostics-3669503-supplementary.pdf]

Table S1. Search strategies for online database

Pubmed		
	**Search terms**	**Total results**
#1	((("Neoplasms"[Mesh])) OR "Carcinoma"[Mesh]) OR "Sarcoma"[Mesh]	4084075
#2	cancer*[Title/Abstract] OR malignanc*[Title/Abstract] OR "malignant neoplasm*"[Title/Abstract] OR carcinoma*[Title/Abstract] OR sarcoma*[Title/Abstract]	3216878
#3	#1 OR #2	5007024
#4	"Mouth"[Mesh]	331468
#5	Oral[Title/Abstract] OR intraoral[Title/Abstract] OR mouth[Title/Abstract]	860,062
#6	#4 OR #5	1123470
#7	"Cone-Beam Computed Tomography"[Mesh]	16,463
#8	"Cone-beam ct"[Title/Abstract] OR cbct[Title/Abstract] OR "cone-beam computed tomography"[Title/Abstract]	23,757
#9	#7 OR #8	26544
#10	#3 AND #6 AND #9	367

Web of Science (WOS)		
	**Search terms**	**Total results**
#1	((((TS=(neoplasm*)) OR TS=(cancer*)) OR TS=(carcinoma*)) OR TS=(sarcoma*)) OR TS=(malignan*)	4,570,509
#2	((TS=(mouth)) OR TS=(oral)) OR TS=(intraoral)	1,036,786
#3	((TS=(cone beam computed tomography)) OR TS=(cbct)) OR TS=(cone-bean ct)	28,077
#4	#1 AND #2 AND #3	203

Scopus		
	**Search terms**	**Total results**
#1	(TITLE-ABS-KEY (neoplasm*) OR TITLE-ABS-KEY (cancer*) OR TITLE-ABS-KEY (carcinoma*) OR TITLE-ABS-KEY (sarcoma*) OR TITLE-ABS-KEY (malignan*))	5,772,694
#2	(mouth) OR ((TITLE-ABS-KEY (oral) OR TITLE-ABS-KEY (intraoral)))	1,684,856
#3	(cone AND beam AND computed AND tomography) OR ((TITLE-ABS-KEY (cbct) OR TITLE-ABS-KEY (cone-beam AND ct)))	42,450

Cochrane library		
	**Search terms**	**Total results**
#1	MeSH descriptor: [Neoplasms] explode all trees	126239
#2	(cancer*):ti,ab,kw OR (carcinoma*):ti,ab,kw OR (sarcoma*):ti,ab,kw OR (malignan*):ti,ab,kw OR (neoplasm*):ti,ab,kw (Word variations have been searched)	265857
#3	#1 OR #2	279152
#4	MeSH descriptor: [Mouth] explode all trees	13834
#5	(oral):ti,ab,kw OR (intraoral):ti,ab,kw OR (mouth):ti,ab,kw (Word variations have been	255120
#6	#4 OR #5	263883
#7	MeSH descriptor: [Cone-Beam Computed Tomography] explode all trees	661
#8	(CBCT):ti,ab,kw OR (Cone-beam CT):ti,ab,kw OR ("cone-beam computed tomography"):ti,ab,kw (Word variations have been searched)	2895
#9	#7 OR #8	2895
#10	#3 AND #6 AND #9	34
#4	((TITLE-ABS-KEY (neoplasm*) OR TITLE-ABS-KEY (cancer*) OR TITLE-ABS-KEY (carcinoma*) OR TITLE-ABS-KEY (sarcoma*) OR TITLE-ABS-KEY (malignan*))) AND ((TITLE-ABS-KEY (mouth) OR TITLE-ABS-KEY (oral) OR TITLE-ABS-KEY (intraoral))) AND ((TITLE-ABS-KEY (cone AND beam AND computed AND tomography) OR TITLE-ABS-KEY (cbct) OR TITLE-ABS-KEY (cone AND beam AND ct)))	531

OVID		
	**Search terms**	**Total results**
#1	exp neoplasms/	4083756
#2	cancer*.mp.	2543941
#3	carcinoma*.mp.	1082525
#4	sarcoma*.mp.	144968
#5	malignan*.mp.	760195
#6	#1 or #2 or #3 or #4 or #5	5085301
#7	exp mouth/	331414
#8	oral.mp.	870152
#9	intraoral.mp.	18563
#10	#7 or #8 or #9	1151651
#11	exp cone-beam computed tomography/	16446
#12	CBCT.mp.	17012
#13	Cone-beam CT.mp.	5669
#14	#11 or #12 or #13	24251
#15	#6 and #10 and #14	312

Table S2. Excluded article based on inclusion and exclusion criteria

Author	Excluded article / Reason of exclusion	Year
**Not primary or secondary studies on the application of CBCT in oral cancer patients (n =62)**		
MacDonald-Jankowski et al 2006	Computed tomography for oral and maxillofacial surgeons. Part 2: Cone-beam computed tomography	2006
Mischkowski et al 2007	Intraoperative navigation in the maxillofacial area based on 3D imaging obtained by a cone-beam device	2007
Zhang et al 2011	Development and validation of a hybrid simulation technique for cone beam CT: application to an oral imaging system.	2011
Anser et al 2011	The task of Cone Beam Computed Tomography in oral cancer	2011
Alongi et a 2012	Prospective phase II trial of cetuximab plus VMAT-SIB in locally advanced head and neck squamous cell carcinoma: Feasibility and tolerability in elderly and chemotherapy-ineligible patients	2012
Rana et al 2012	Advances and innovations in computer-assisted head and neck oncologic surgery.	2012
Ahmad et al 2012	Application of cone beam computed tomography in oral and maxillofacial surgery.	2012
Hunt et al 2013	TU-E-141-05: Dosimetric and Anatomical Evaluation of Normal Tissue Changes During IMRT Radiotherapy for Head and Neck Cancer	2013
Li et al 2014	Magnetic Resonance Imaging for Diagnosis of Mandibular Involvement from Head and Neck Cancers: A Systematic Review and Meta-Analysis	2014
Fiorentino et al 2014	Head and neck intensity modulated radiotherapy parotid glands: time of re-planning.	2014
Li et al 2014	Computed Tomography for the Diagnosis of Mandibular Invasion Caused by Head and Neck Cancer: A Systematic Review Comparing Contrast-Enhanced and Plain Computed Tomography	2014
Khan et al 2015	Diagnosis and management of osteonecrosis of the jaw: a systematic review and international consensus.	2015
Suomalainen et al 2015	Rapid prototyping modelling in oral and maxillofacial surgery: A two year retrospective study.	2015
Li et al 2015	Emission Computed Tomography for the Diagnosis of Mandibular Invasion by Head and Neck Cancers: A Systematic Review and Meta-Analysis	2015
Deshpande et al 2015	Osteoradionecrosis of the mandible: Through a radiologist's eyes	2015
Mahdian et al 2016	Tissue characterization using optical coherence tomography and cone beam computed tomography: a comparative pilot study	2016
Wiant et al 2016	A prospective evaluation of open face masks for head and neck radiation therapy	2016
Kamitani etl al 2016	Feeding Arteries of Primary Tongue Cancers on Intra-arterial Infusion Chemotherapy.	2016

Tian et al 2017	Reconstruction of Mandible: A Fully Digital Workflow From Visualized Iliac Bone Grafting to Implant Restoration	2017
Ma et al 2017	Intraoperative image guidance in transoral robotic surgery: A pilot study	2017
Liang et al 2017	Application of Combined Osteotomy and Reconstruction Pre-Bent Plate Position (CORPPP) Technology to Assist in the Precise Reconstruction of Segmental Mandibular Defects	2017
Zhang et al 2018	Jaw Segmentation from CBCT Images	2018
Boeddinghaus et al 2018	Trends in maxillofacial imaging	2018
Van Baar et al 2018	Accuracy of computer-assisted surgery in mandibular reconstruction: A systematic review	2018
Hoving et al 2018	Optimisation of three-dimensional lower jaw resection margin planning using a novel Black Bone magnetic resonance imaging protocol	2018
Walton et al 2019	Medication related osteonecrosis of the jaw in osteoporotic vs oncologic patients-quantifying radiographic appearance and relationship to clinical findings	2019
Bartella et al 2019	Virtual reality in preoperative imaging in maxillofacial surgery: implementation of “the next level”?	2019
Appendino et al 2019	Are intraoral customized stents still necessary in the era of Highly Conformal Radiotherapy for Head & Neck cancer? Case series and literature review	2019
Alawy et al 2020	The virtual surgery to reconstruct the mandible using digital images	2020
Merema et al 2020	Novel finite element-based plate design for bridging mandibular defects: Reducing mechanical failure	2020
Liang et al 2020	Use of artificial intelligence to recover mandibular morphology after disease.	2020
Glas et al 2021	Augmented Reality Visualization for Image-Guided Surgery: A Validation Study Using a Three-Dimensional Printed Phantom	2021
Patel et al 2021	The use of pentoxifylline, tocopherol and clodronate in the management of osteoradionecrosis of the jaws	2021
Franzese et al 2021	Metastasis-directed stereotactic body radiation therapy in the management of oligometastatic head and neck cancer	2021
Özseven et al 2021	Evaluation of patient organ doses from kilovoltage cone-beam CT imaging in radiation therapy	2021
Weppler et al 2021	Patient-Reported Outcomes-Guided Adaptive Radiation Therapy for Head and Neck Cancer	2021
Cuéllar et al 2021	Virtual surgical planning, stereolitographic models and cad/cam titanium mesh for three-dimensional reconstruction of fibula flap with iliac crest graft and dental implants	2021
Abhinaya et al 2022	Clinical practice guidelines for radiographic assessment in management of oral cancer	2022
Guss et al 2022	Head and Neck Radiation Therapy: From Consultation to Survivorship and Future Directions	2022
Mishra et al 2022	Recent advancements in imaging of oral cancer	2022
Bø et al 2022	Radiotherapy of tongue cancer using an intraoral stent: a pilot study	2022

Nel et al 2022	Radiological spectrum of metastasis to the oral and maxillofacial region	2022
Jonovic et al 2022	Evaluation of radiodensity and dimensional stability of polymeric materials used for oral stents during external beam radiotherapy of head and neck carcinomas.	2022
Carretero et al 2022	Virtual Surgical Planning and Customized Subperiosteal Titanium Maxillary Implant (CSTMI) for Three Dimensional Reconstruction and Dental Implants of Maxillary Defects after Oncological Resection: Case Series	2022
Hung et al 2022	Current Applications of Deep Learning and Radiomics on CT and CBCT for Maxillofacial Diseases.	2022
Spenkelink et al 2022	Image-guided procedures in the hybrid operating room: A systematic scoping review	2022
Tagliamento et al 2022	Denosumab related osteonecrosis of the jaw: Unusual pattern with periosteal reaction	2022
Nilsson et al 2022	Ultrasound accurately assesses depth of invasion in T1-T2 oral tongue cancer	2022
Boopathi et al 2023	Imaging Pitfalls and Diagnostic Inhibitions in Various Advanced Head and Neck Imaging Modalities—Diagnostician's Perspective	2023
Mortazavi et al 2023	Evaluation of Radiographic Features and Clinical Signs of Maxillofacial Malignancies in Patients Referred to Mashhad Dental School During 2003-2017	2023
Alexidis et al 2023	Investigating Predictive Factors of Dysphagia and Treatment Prolongation in Patients with Oral Cavity or Oropharyngeal Cancer Receiving Radiation Therapy Concurrently with Chemotherapy	2023
Chappuis et al 2023	An aggressive pregnancy epulis with extensive alveolar bone resorption and tooth displacement	2023
Lee et al 2023	Bisphosphonates and Their Connection to Dental Procedures: Exploring Bisphosphonate-Related Osteonecrosis of the Jaws	2023
Liu et al 2024	Automatic classification and segmentation of multiclass jaw lesions in cone-beam CT using deep learning.	2024
Saba et al 2024	Intensity-Modulated Reirradiation Therapy with Nivolumab in Recurrent or Second Primary Head and Neck Squamous Cell Carcinoma: A Nonrandomized Controlled Trial	2024
Akkoyunlu et al 2024	Radiological Features of Rare Non-odontogenic Lesions of the Jaws	2024
Rokhshad et al 2024	Deep learning for diagnosis of head and neck cancers through radiographic data: a systematic review and meta-analysis	2024
Kale et al 2024	Is it time to move from the Unidimensional RECIST 1.1 Response Assessment Criteria to a Volumetric Evaluation in the Present Era of Image-based Oncology? An Evaluation in Locally Advanced Head Neck Cancers Undergoing Treatment	2024
Haderlein et al 2024	De-intensification of postoperative radiotherapy in head and neck cancer irrespective of human papillomavirus - results of a prospective multicenter phase II trial (DIREKHT Trial)	2024
Eu et al 2024	Evaluation of a 3D Printed Silicone Oral Cavity Cancer Model for Surgical Simulations	2024

Peters et al 2024	Development of a universal cutting guide for raising deep circumflex iliac artery flaps.	2024
Santhish et al 2024	Sivan classification system for diagnosis of jaw lesions based on visual volumetric analysis of 3-dimensional cone-beam computed tomographic images.	2024
**Conference abstract, letters, author's personal opinions, book chapters, case reports and editorials (n=11)**		
Closmann et al 2007	The use of cone beam computed tomography as an aid in evaluating and treatment planning for mandibular cancer.	2007
Buchanan et al 2015	Unusual presentation of extranodal diffuse large B-cell lymphoma in the head and neck: Description of a case with emphasis on radiographic features and review of the literature	2015
Locarevic et al 2016	Bilateral numb chin syndrome as a symptom of breast cancer metastasis in the mandible: A case report and discussion on the usefulness of cone-beam computed tomography to assess bone involvement in oral cancer	2016
Mintline et al 2022	The Synergistic Role of 2D and 3D Imaging in Evaluating Tumors of the Jaws: A Case Report of Diffuse Large B-Cell Lymphoma of the Mandible	2022
Dohopolski et al 2022	Dosimetric Impact of Simulated Daily Adaptive Radiotherapy with Significantly Reduced Setup Margins in the Definitive Treatment of Head and Neck Cancer	2022
Ali et al 2022	Osteolytic Lesion of the Maxilla in an Undiagnosed Multiple Myeloma Patient Identified Incidentally by Cone Beam Computed Tomography.	2022
Zisis 2023	Oral Squamous Cell Carcinoma (OSCC) Imitates Denosumab-Induced Osteonecrosis of the Mandibular Alveolus: A Diagnostic Challenge.	2023
Wang et al 2024	Solution for the External Contour Changes in Cone Beam Computed Tomography-Guided On-demand Online Adaptive Radiotherapy for a Patient With Very Advanced Head and Neck Cancer: A Technical Case Report.	2024
Polard et al 2024	When Maxillofacial CBCT Permits Fortuitously to Diagnose Primary Non-Hodgkin's Lymphoma: A Case Report	2024
Markovic Vasiljkovic et al 2024	Chondrosarcoma of the Alveolar Process of the Mandible Initially Suspected to Be a Periodontal Lesion.	2024
Nair et al 2024	Role of cone-beam computed tomography in evaluating osseous invasion from oral squamous cell carcinoma.	2024
**Other head and neck cancer (n=38)**		
Heiland et al 2008	Navigated implantation after microsurgical bone transfer using intraoperatively acquired cone-beam computed tomography data sets.	2008
Mechalakos et al 2009	The effect of significant tumor reduction on the dose distribution in intensity modulated radiation therapy for head-and-neck cancer: a case study.	2009
Yang et al 2010	Utility of megavoltage fan-beam CT for treatment planning in a head-and-neck cancer patient with extensive dental fillings undergoing helical tomotherapy	2010

Peroni et al 2012	Automatic segmentation and online virtualCT in head-and-neck adaptive radiation therapy.	2012
Ho et al 2012	Monitoring dosimetric impact of weight loss with kilovoltage (kV) cone beam CT (CBCT) during parotid-sparing IMRT and concurrent chemotherapy.	2012
Reaungamornrat et al 2013	A Gaussian mixture + demons deformable registration method for cone-beam CT-guided robotic transoral base-of-tongue surgery	2013
Fiorentino et al 2013	Cone-beam computed tomography dose monitoring during intensity-modulated radiotherapy in head and neck cancer: parotid glands.	2013
Fu et al 2014	Importance of CBCT setup verification for optical-guided frameless radiosurgery.	2014
Liu et al 2015	Augmented reality and cone beam CT guidance for transoral robotic surgery	2015
Liu et al 2015	Intraoperative image-guided transoral robotic surgery: pre-clinical studies.	2015
Korpics et al 2016	Metal Artifact Reduction in Cone-Beam Computed Tomography for Head and Neck Radiotherapy	2016
Broggi et al 2017	A Comparative Evaluation of 3 Different Free-Form Deformable Image Registration and Contour Propagation Methods for Head and Neck MRI: The Case of Parotid Changes During Radiotherapy.	2017
Duffy et al 2017	The dilemma of parotid gland and pharyngeal constrictor muscles preservation-Is daily online image guidance required? A dosimetric analysis.	2017
Veresezan et al 2017	Adaptive radiation therapy in head and neck cancer for clinical practice: state of the art and practical challenges	2017
Woodford et al 2019	Contrast enhanced oesophageal avoidance for stereotactic body radiotherapy: barium vs Gastrografin	2019
Lee et al 2020	Predictive dose accumulation for HN adaptive radiotherapy.	2019
Wagenaar et al 2019	Composite minimax robust optimization of VMAT improves target coverage and reduces non-target dose in head and neck cancer patients.	2019
Simpione et al 2020	Tomographic study of Jaw bone changes in patients with bisphosphonate-related osteonecrosis.	2020
Dai et al 2021	Head-and-neck organs-at-risk auto-delineation using dual pyramid networks for CBCT-guided adaptive radiotherapy.	2020
Feitosa et al 2020	Bisphophonate alterations of the jaw bones in individuals with multiple myeloma.	2020
Lim et al 2021	Can the Risk of Dysphagia in Head and Neck Radiation Therapy Be Predicted by an Automated Transit Fluence Monitoring Process During Treatment? A First Comparative Study of Patient Reported Quality of Life and the Fluence-Based Decision Support Metric.	2021
Lentzen et al 2021	Bisphosphonate application and volumetric effects on MRONJ lesions.	2021

Imaizumi et al 2022	Cone beam computed tomography with oral contrast for accurate diagnosis and surgical planning of pharyngeal leakage and fistula: a case series	2022
Hirotaki et al 2022	Detection of anatomical changes using two-dimensional x-ray images for head and neck adaptive radiotherapy.	2022
Jiang et al 2022	Comparison of an in-house hybrid DIR method to NiftyReg on CBCT and CT images for head and neck cancer.	2022
Muttanahally et al 2023	Radiographic Evaluation of Medication-Related Osteonecrosis of the Jaw (MRONJ) With Different Primary Cancers and Medication Therapies.	2023
Gong et al 2023	Evaluation of Dose Calculation Based on Cone-Beam CT Using Different Measuring Correction Methods for Head and Neck Cancer Patients.	2023
Smolders et al 2024	The influence of daily imaging and target margin reduction on secondary cancer risk in image-guided and adaptive proton therapy.	2024
Arya et al 2024	An institutional protocol including socket alveoplasty and primary closure following dental extractions for patients with an elevated risk of developing medication-related osteonecrosis of the jaw.	2024
Shahid et al 2024	Maxillary interim obturator prosthesis fabrication for a patient with limited mouth opening with a digital approach: A clinical report.	2024
Dohopolski et al 2024	In silico evaluation and feasibility of near margin-less head and neck daily adaptive radiotherapy.	2024
Aristophanous et al 2024	Clinical Experience With an Offline Adaptive Radiation Therapy Head and Neck Program: Dosimetric Benefits and Opportunities for Patient Selection.	2024
Lindfors et al 2024	Is cone-beam computed tomography (CBCT) an alternative to plain radiography in assessments of dental disease? A study of method agreement in a medically compromised patient population.	2024
Nuchsirikulaphong et al 2024	The impact of air cavity changes on adaptive plan quality of the intensity modulated radiation therapy in head and neck cancer for magnetic resonance linear accelerator treatment	2024
Wang et al 2024	Impact of Manual Contour Editing on Plan Quality for Online Adaptive Radiation Therapy for Head and Neck Cancer.	2024
Gracea et al 2025	Alveolar socket surface area as a local risk factor for MRONJ development in oncologic patients on polypharmacy.	2025
Kil et al 2025	Pharyngeal Constrictor-Sparing Salvage Stereotactic Body Radiation Therapy With Tongue-Out for In-Field Recurrence After Definitive Radiation Therapy for Head and Neck Cancer: Guide to Tongue-Out Radiation Therapy.	2025
Yang et al 2025	Effectiveness of personalized open-face mask combined with styrofoam fixation in radiotherapy treatment of head and neck cancers: a prospective randomized controlled trial.	2025
**Unavailability to obtained full text (n=9)**		

Li et al 2008	SU-GG-J-95: Feasibility to Use Daily Cone-Beam-CT to Determine Dose-Surface Histogram of Oral Mucosa in Radiotherapy of H&N Cancer	2008
Morimoto 2009	New Trends and Advances in Oral and Maxillofacial Imaging	2009
Borumandi et al 2015	Anti-resorptive drugs and their impact on maxillofacial bone among cancer patients	2015
Daly et al 2019	Non-contact fluorescence tomography with a cone-beam CT surgical guidance system	2019
Dai et al 2021	Synthetic MRI-aided multi-organ segmentation in head-and-neck cone beam CT	2021
Patel et al 2022	Oral Cancer Detection and Diagnosis: A New Frontier in Artificial Intelligence	2022
Abdalla-Aslan et al 2023	Radiographic findings of space-occupying lesions in sialo-CBCT of the major salivary glands.	2023
Rocchetti et al 2023	Digital workflow for the intraoral removable prosthesis of head and neck cancer patients	2023
Hirotaki et al 2023	Impact of Anatomical Position Errors on Dose Distribution in Head and Neck Radiotherapy and Robust Image Registration Against Anatomical Changes	2023
	**Article not in English language (n=4)**	
Bombeccari et al 2015	Diagnostic accuracy of cone beam computed tomography (CBCT) to detect bone invasion by oral carcinoma	2015
Fan et al 2022	Accuracy of detachable 3D-printed Stent placement in radiotherapy for oral Cancer patients	2022
Husain et al 2024	Challenges in the diagnosis and management of soft tissue tumors of the oral cavity.	2024
Carsuzaa et al 2024	Prevention, diagnosis and management of osteoradionecrosis: Where do we stand?	2024
